# Elements of Physics, or Natural Philosophy, General and Medical, &c.

**Published:** 1827-07

**Authors:** 


*Elements of Physics, or Natural Philosophy, general and medical, explained independently of Technical Mathematics.* By N. Arnott, M.D. of the Royal College of Physicians. Octavo, pp. 611. 1827.

We think there can be but one opinion as to the fact, that a general and moderate acquaintance with the laws of natural philosophy is not only ornamental but highly useful to the medical practitioner, of whatever denomination. Medical men mix in good society—and in good society general knowledge prevails. They are expected to be able to converse on general subjects of literature and science—and it would be indecent for them to introduce subjects purely medical, though they may occasionally answer queries of that nature. Were there no other reasons than these for the study of natural philosophy, they would be quite sufficient: but there are many other reasons. Mechanics, hydraulics, gravitation, attraction, pneumatics, and many other branches of natural knowledge are more or less intimately blended with physiology, pathology, and even therapeutics, so as to render a general acquaintance with them *almost necessary*—and, at all events, highly useful. If this be granted, and we think it must be granted, the next question is this: has Dr. Arnott offered greater facilities to the medical student, for the acquisition of this general knowledge of natural philosophy, than he could find in any other work of equal dimensions? We answer, that we believe he has. By great attention to arrangement, and to avoiding, as much as possible, all abstruse reasonings and technicalities—by a pleasing and familiar style—and by seductive illustrations, he has rendered the path of science (hitherto beset with thorns and all kinds of difficulties) a promenade of pleasure. We do not say that the work before us is calculated for those who have made, or who mean to make, deep researches into each subject of natural philosophy:—but we mean to say, that the work is well adapted for the general reader and the medical student, who only want or wish for as much knowledge of physics as may prove useful and ornamental in their own proper pursuits through life. This is all, we apprehend, that Dr. Arnott has aimed at, and we think his undertaking will be crowned with success.

To attempt any analysis of a work of this description would be perfectly ridiculous; and therefore we must content ourselves with two or three extracts from an admirably written preface, by which the reader will form some idea of the *manner* in which our author treats his subjects.

*First Extract.*

"*Physics.* The laws of *Physics* govern every phenomenon of nature in which there is any sensible change of place, and they are themselves the sole causes of the greater part of all the phenomena. They *regulate* also those which *originate* from chemical action, and from the action of life.—The great Physical truths are now reduced to four, and are referred to by the words *atom*, *attraction*, *repulsion*, and *inertia*. It gives an astonishing, but true idea of the nature and importance of methodical *Science*, to be told that a man, who understands these words, *viz.* how the *atoms* of matter *attract*, and cling together to form masses, which are solid, liquid, or aeriform, according to the quantity of *repelling* heat among them, and which, owing to their *inertia* or stubbornness, gain and lose motion, in exact proportion to the force of attraction or repulsion acting on them, understands the greater part of the phenomena of nature; but such is the fact! *Solid* bodies, existing in conformity with these truths, exhibit all the phenomena of *Mechanics; Liquids* exhibit those of *Hydrostatics* and *Hydraulics; Airs*, those of *Pneumatics;* and so forth, as seen in the table of heads below, page xix. And the whole of this volume is merely a list of the most interesting physical phenomena, arranged in classes under these heads."—*Pref.* xv.

*Second Extract.*

"*Mind.* The most important part of all science, is the knowledge which man has obtained of the laws which govern the operations of his own MIND. This department stands eminently distinct from the others, on several accounts. Unlike that of *organic life*, which could not be understood until physics and chemistry had been previously investigated, this attained extraordinary perfection in a very early age, when the others had scarcely begun to exist. We need only refer to the writings of the Greek philosophers, in proof of the assertion. The brilliant discoveries, however, were reserved for the moderns, as will occur to most readers, on perusing in the table below the divisions of the subject, and recollecting the honoured names which are now associated with each. It is truly admirable to see the modern analysis deducing from a few simple laws of mind, all the subordinate departments, just as it deduces mechanics, hydrostatics, &c. from the laws of physics.—It is to be remarked, that the laws of mind which man can discover by reason, are not laws of independent mind, but of mind in connexion with body and influenced by the bodily condition. It has been believed by many, that the nature of mind separate from body, is to be at once all-knowing and intelligent. But mind connected with body, can only acquire knowledge slowly, through the bodily organs of sense, and more or less perfectly, according as these organs and, the central brain are perfect. A human being born blind and deaf, and therefore, remaining dumb, as in the extraordinary case of the boy Mitchell, which excited so much attention some years ago, grows up to resemble an automaton: and an originally mis-shapen or deficient brain causes idiotcy for life. Childhood, maturity, dotage, which have such differences of bodily powers, have corresponding differences of mental faculty; and as no two bodies, so no two minds as manifested externally, are quite alike. Fever or a blow on the head will change the most gifted individual into a maniac; and most cases of madness and of eccentricity can now be traced to a peculiar state of the brain.—Man has a conviction inseparable from his very being, that his soul is something distinct from his body, and awaiting other destinies: but, independently of Revelation, his notions on the subject remain very vague, as is shewn in the laborious reasonings of the ancient heathen philosophers." xviii.

*Third Extract.*

"And besides all these, and other uses, Physics is an important foundation of the healing art. The medical man, indeed, is the engineer pre-eminently; for it is in the animal body that true perfection and the greatest variety of mechanism are found. Where is there, to illustrate *Mechanics*, a system of levers, and hinges, and moving parts, like the limbs of an animal body; where such an *hydraulic* apparatus, as in the heart and blood-vessels; such a pneumatic apparatus, as in the breathing chest; such *acoustic* instruments, as in the ear and larynx; such an *optical* instrument, as in the eye; in a word, such mechanical variety and perfection, as in the whole of the visible anatomy! All these structures, the medical man, of course, should understand, as a watchmaker knows the parts of the machine about which he is employed. The latter, unless he can discover where a pin is loose, or a wheel injured, or a particle of dust adhering, or oil wanting, &c. would ill succeed in repairing an injury: and so also of the ignorant medical man in respect to the human body. Yet will it be believed, that there are medical men who neither understand mechanics, nor hydraulics, nor pneumatics, nor optics, nor acoustics, beyond the merest routine; and that systems of medical education are put forth at this day which do not even mention the department of *Physics!*" xxviii.

The work is divided into five chapters, embracing Somatology and Dynamics—Mechanics—Hydrodynamics, (including Hydrostatics, Pneumatics, Hydraulics, and Acoustics)—Phenomena of imponderable matter, (Caloric, Optics, Electricity, &c.) —and Astronomy. Under each chapter are ranged the illustrations afforded by the Animal Economy, viz. Animal and Medical Physics. The whole is illustrated by numerous woodcuts, and rendered extremely easy of comprehension, even by the general reader, with but a moderate education.

Were we disposed, we think we could make some objections to certain portions of Dr. Arnott's Medical Physics, while treating of the circulation of the blood. But upon the whole, our author comes nearer to our own views of this important phenomenon, than any author with whom we are acquainted. We are vain enough to think that Dr. A. has adopted some opinions and illustrations broached in this Journal, on the subject of the circulation, although the nature of his work precluded all reference to the sources of his information.

Dr. A. has criticised Dr. Barry's doctrines; but as that gentleman is well able to defend himself, we shall not take any further notice of the controversy.

We need scarcely add, in conclusion, that we recommend, in the strongest terms, the work before us, to every medical student—and to every practitioner who has not paid sufficient attention to the laws of physics, in the early part of his education.

